# Gender differences in association between expiratory dynamic airway collapse and severity of obstructive sleep apnea

**DOI:** 10.1007/s00330-023-10322-x

**Published:** 2023-11-14

**Authors:** Soriul Kim, Ki Yeol Lee, Ali Tanweer Siddiquee, Hyeon Jin Kim, Hye Ryeong Nam, Chang Seop Ko, Nan Hee Kim, Chol Shin

**Affiliations:** 1 Institute for Human Genomic Study, College of Medicine, Korea University, Seoul, Republic of Korea; 2 Division of Sleep Medicine, Department of Medicine, University of Pennsylvania, Philadelphia, PA USA; 3 Department of Radiology, Korea University Ansan Hospital, #123, Jeokgeum-Ro, Danwon-Gu, Ansan, 15355 Republic of Korea; 4 Department of Radiology, Korea University Guro Hospital, Seoul, Republic of Korea; 5 Department of Neurology, Korea University Ansan Hospital, Ansan, Republic of Korea; 6 Division of Endocrinology and Metabolism, Department of Internal Medicine, Korea University Ansan Hospital, Ansan, Republic of Korea; 7 Biomedical Research Center, Korea University Ansan Hospital, Ansan, Republic of Korea; 8 Division of Respiratory and Critical Care, Department of Internal Medicine, Korea University Ansan Hospital, #123, Jeokgeum-Ro, Danwon-Gu, Ansan, 15355 Republic of Korea

**Keywords:** Obstructive sleep apnea, Expiratory dynamic tracheal collapse, Gender differences, Chest multidetector computed tomography

## Abstract

**Objectives:**

Repetitive unbalances and tensions generated by inspiratory efforts against an obstructive upper airway during sleep predispose the development of expiratory central airway collapse. In addition, structures of the upper airway between men and women have differences and could be the reasons for differences in obstructive sleep apnea (OSA) prevalence between genders. The present study aimed to evaluate the association between parameters of expiratory dynamic tracheal collapse measured using chest multidetector CT and objectively measured OSA severity between men and women.

**Materials and methods:**

A total of 901 participants who underwent chest CT and overnight in-home polysomnography from the Korean Genome and Epidemiology Study were cross-sectionally analyzed (women: 46.2%). The participants were divided into three groups based on OSA severity by apnea–hypopnea index (AHI). Multivariate linear regression analysis was performed to determine the effects of central airway collapse after adjustment for cardiovascular-related covariates.

**Results:**

In a multivariate analysis, percentages of expiratory lumen structure reductions involving area, diameter, and perimeter were associated with AHI (all *p* values < 0.05) and with OSA severity (moderate-to-severe OSA than no OSA: *β* = 3.30%, *p* = 0.03; *β* = 2.05%, *p* = 0.02; *β* = 1.97%, *p* = 0.02, respectively) in women, whereas men had only a greater percentage of expiratory wall thickness reduction in moderate-to-severe OSA than no OSA (*β* = 0.72%, *p* = 0.003). In addition, women with both mild OSA and moderate-to-severe OSA had higher expiratory tracheal collapse than men without OSA, and a moderate effect of sex was observed (*p* for interaction = 0.007).

**Conclusion:**

The expiratory dynamic tracheal collapse was independently associated with severity of OSA in women than in men.

**Clinical relevance statement:**

Differences of pharyngeal structures and inherent features of airways by genders may affect the dissimilarities in vulnerability to sleep apnea between men and women.

**Key Points:**

*• The expiratory dynamic tracheal collapse was independently associated with severity of OSA in women than in men.*

*• Women with over mild OSA had higher expiratory tracheal collapse* *than men without OSA, and moderate effect of sex was observed.*

*• Structural differences of airway may affect differences in susceptibility of sleep apnea between genders.*

**Supplementary Information:**

The online version contains supplementary material available at 10.1007/s00330-023-10322-x.

## Introduction

Obstructive sleep apnea (OSA) is a common pattern of sleep disordered breathing characterized by repeated partial or complete narrowing and closure of the upper airway, leading to intrathoracic pressure changes, intermittent hypoxia, and sleep fragmentation [1, 2]. Epidemiological studies in the general population reported that the male:female ratio of OSA syndrome is 2–4:1 [3, 4], but these figures may still underestimate the prevalence of OSA in women. In previous studies, compared to men, women have different symptoms, different severity of symptoms, or they underreport their symptoms [5, 6]. In terms of symptoms in women, they are less likely to recount witnessed apnea or snoring but are more likely to complain of insomnia, nightmares, morning headaches, and daytime fatigue, compared to men [7, 8]. Thus, women may be misdiagnosed as having depression and have a delayed diagnosis of OSA more frequently than men [7]. The consequences may be similar or worse with women suffering greater risk for OSA-affected health outcome even though the prevalence and severity of OSA may be lower in women [9]. Therefore, it is important to understand what gender-related symptoms or inherent features of airways exist that might contribute to the prevalence of OSA, and then we could be looking for which treatment options provide best outcomes for women.

Meanwhile, expiratory central airway collapse (ECAC) has been described to involve tracheobronchomalacia, characterized by weakness of the tracheobronchial supporting elements including myoelastic and cartilages, and excessive dynamic airway collapse, defined as extreme bulging of the posterior membrane into the airway lumen during expiration [10, 11]. Paired end-inspiratory and dynamic expiratory computed tomography (CT) scans have been shown to be an efficient and non-invasive method for diagnosing tracheobronchomalacia [12]. The severity of ECAC can be determined as the percentage of anterior–posterior narrowing of the tracheal or main bronchial lumen/wall during forceful expiration or as the percentage of reduction in tracheal or bronchial cross-sectional lumen surface area [10–13], which can be obtained either through direct observation of the airway with bronchoscopy or through dynamic expiratory imaging with multidetector CT [14]. In adults, the severity of expiratory collapse is proposed as mild (70–80%), moderate (81–90%), or severe (> 90%), but the true figuring out of prevalence of ECAC is less than perfect, because this is an underestimated condition with overlapping comorbidities in patients and a lack of standardized diagnostic criteria. Patients are often asymptomatic or may have nonspecific symptoms [11].

Previous reports have shown that ECAC is associated with respiratory diseases, such as asthma, chronic obstructive pulmonary disease, and upper gastrointestinal disorders [10, 14]. A variety of inflammatory, repeated infections, and traumatic processes are also known to contribute to ECAC, leading to destruction of cartilage or contributing to expiratory airway collapse [11]. Additionally, OSA is considered one of the causes of ECAC based on pathophysiological principles. There are many factors that contribute to alterations in airway diameter leading to recurrent airway obstruction during sleep including size and stiffness of the airway lumen, and the pressure gradient across the airway wall [9]. Repetitive imbalances and tensions generated by inspiratory efforts against an obstructive upper airway during sleep could promote the development of ECAC [15, 16]. Meanwhile, properties of the upper airway between men and women are shown to have clear differences, so then could be reasons for dissimilar OSA prevalence between genders. Although OSA or sleep-disordered breathing has been associated with ECAC in previous case–control studies [17, 18], there is limited data and it is unclear whether gender-related differences might contribute to OSA prevalence [15, 19].

The aim of this study was to investigate whether the degree of dynamic airway collapsibility, one of the characteristics in ECAC, as noninvasively measured using chest multidetector CT, would be associated with objectively measured OSA severity between men and women. We analyzed paired inspiratory-expiratory chest CT scans of participants from a general population-based cohort to determine the degree of dynamic airway collapse by OSA severity.

## Materials and methods

### Study design and population

The study protocol was approved by the institutional ethics committee of the Korea University Ansan Hospital and written informed consent was obtained from all participants (IRB Nos. 2006AS0045 and 2016AS0043).

**Fig. 1:**
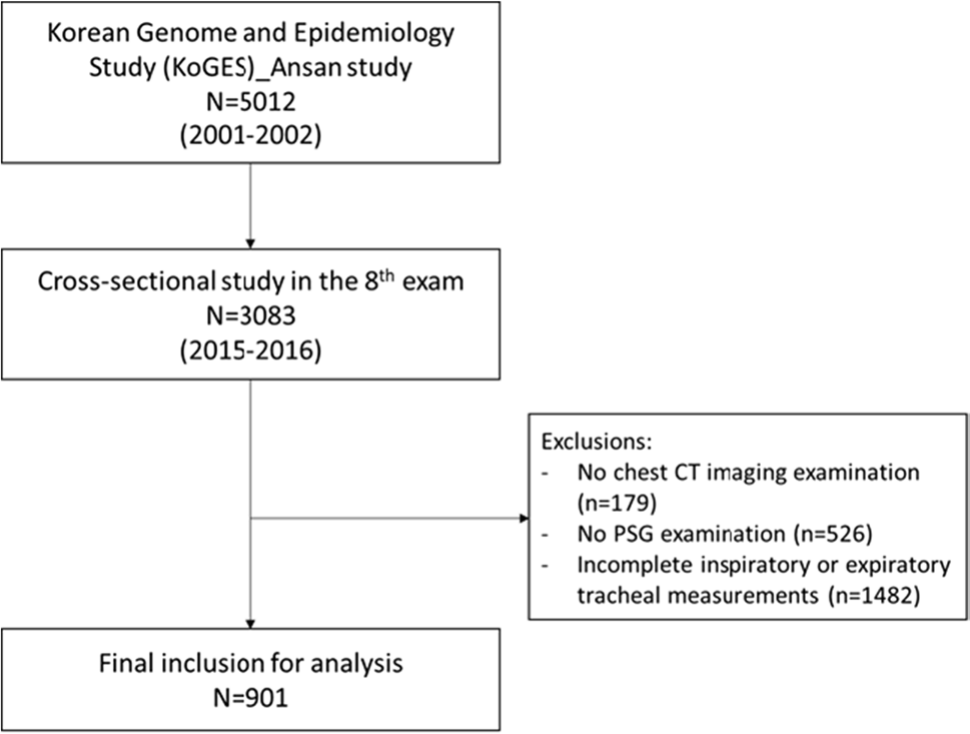
Flow diagram of participants in the KoGES_Ansan study, 2001–2016. KoGES, Korean Genome and Epidemiology Study; CT, computed tomography; PSG, polysomnography

This study included participants from a population-based cohort comprising Korean men and women aged 39–70 years. All study participants were part of the Korean Genome and Epidemiology Study, which is an ongoing prospective investigation. Detailed information on participant recruitment is available elsewhere [20–22]. Briefly, a total of 5012 participants from Ansan, South Korea, were examined at baseline between 2001 and 2002. The cohort participants had a questionnaire-based interview and health examination, and biospecimens were collected by health professionals. The questionnaire included demographic characteristics, medical history, lifestyle, and sleep-related parameters. Additional details can be found in Supplementary information. Follow-up examinations were performed biennially during scheduled site visits. The eighth examination was completed between March 2015 and December 2016 and a total of 3083 participants attended. Of these participants, 2378 underwent chest CT and overnight in-home polysomnography (PSG). In total, 2157 of the 2378 participants have been previously reported [20]. This prior study dealt with the association between OSA and the presence of subclinical systemic atherosclerosis. From this sample, we further excluded participants with incomplete inspiratory or expiratory tracheal measurements (*n* = 1477). Finally, 901 individuals were analyzed (Fig. 1).

### Polysomnography

Full-night PSG recordings with Embletta X100 were performed at the participants’ home (Embletta X-100, Embla Systems). The PSG recording signs consisted of one electroencephalographic channel (C4), left or right electrooculogram, chin muscle electromyograms, electrocardiograms, chest and abdominal respiratory effort bands, pressure transducer airflow sensor, snore sensor and body position sensors, and pulse oximetry. A trained professional connected the device to the individual at bedtime; data were collected the morning after the unattended overnight recording; and the PSG results were manually scored according to standard criteria [23]. Additional details can be found in Supplementary information. The apnea–hypopnea index (AHI) was calculated by averaging the total numbers of obstructive apnea and hypopnea events per hour of sleep, and severity of OSA was defined according to AHI, as follows: no OSA (AHI < 5), mild OSA (5 ≤ AHI < 15), and moderate-to-severe OSA (15 ≤ AHI).

### CT imaging protocol and measurements

The details of the procedures for CT scans acquisition, scanner quality control, and imagery interpretation have been previously described [24, 25]. The CT scans were acquired in the supine position during end-inspiratory and expiratory breath holding using a commercial 64-channel multidetector CT (Brilliance 64, Philips Healthcare) following a standardized protocol. Scanning parameters were held constant as follows: 64 × 0.625 mm detector configuration, 120 kV (peak), 100 mAs, and a section thickness of 0.625 mm without intravenous contrast material. Tube current modulation was not applied.

**Fig. 2:**
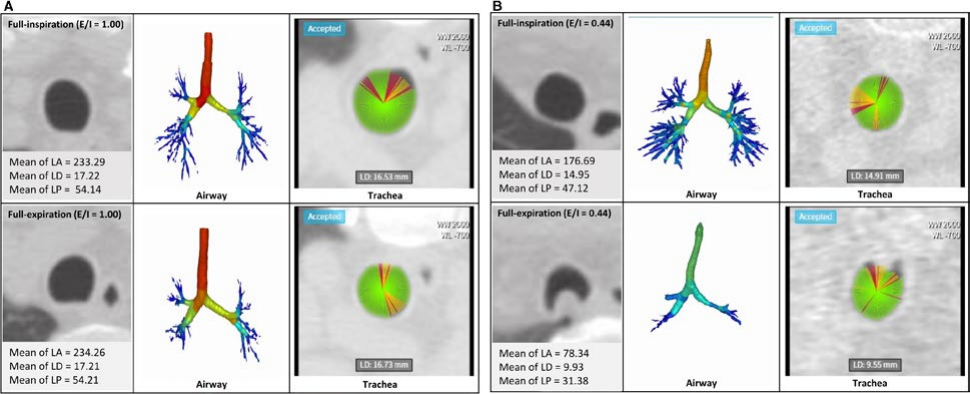
Quantitative CT measurement of trachea. Once airways were extracted and target points in the trachea were selected, quantitative tracheal parameters such as luminal diameter and wall thickness were automatically measured. Full-expiration and full-inspiration images in a healthy participant (**A**) showed the normal range of the change in shape and in luminal area, diameter and perimeter; expiratory-to-inspiratory (*E*/*I*) ratio of tracheal lumen area = 1.00. Full-expiration and full-inspiration images in a participant with expiratory central airway collapse (**B**) showed the change in shape and decrease in tracheal luminal area, diameter, and perimeter;* E*/*I* ratio of tracheal lumen area = 0.44. LA, tracheal luminal area; LD, tracheal luminal diameter; LP, tracheal luminal perimeter

Tracheal measurements were performed using commercial software (Aview, Coreline Soft) to automatically segmentation (Fig. 2) [26, 27]. For the geometric measurements of the airway lumen and wall, we extracted 10 equally spaced cross-sectional images from the trachea (online supplementary Figure E1A). From one selected airway image, 120 profiles measuring the wall thickness along the airway wall are obtained (online supplementary Figure E1B). In each profile, we extracted three values: lumen diameter (*LD*), wall thickness, and peak position for the wall. The method of measuring airway diameter was also the same, measuring tracheal diameter using 120 profiles for each of the 10 selected CT images (online supplementary Figure E1C). The lumen diameter and wall thickness of the airway were analyzed at each location, and the average value was used as a representative analysis value. Parameters such as lumen diameter and wall thickness of trachea were obtained from each profile using the full width at half-maximum (FWHM) method. We had calculated mean and standard deviation of the measured values. After then, profiles that differ less than 1.2σ from mean of lumen radii were included, and profiles that differ more than 1.2σ from mean of lumen radii were excluded. Each sample was accepted or rejected based on its profile and measured values, and the mean value of the accepted samples became the representative value of the airway branch. The formula for obtaining the luminal perimeter (*LP*) was as follows: $LP=2\sqrt{LA\times \pi }$ (*LP*, lumen perimeter; *LA*, lumen area). The tracheal lumen cross-sectional surface areas were measured on end-inspiratory and dynamic expiratory CT scans and these measurements used to calculate the percentage of tracheal collapsibility with following equation:

$$Percentage\, of\, tracheal\, luminal\, collapse=[1-\left(\frac{tracheal\, luminal\, area\, on\, dynamic\, expiration}{tracheal\, luminal\, area\, at\, end\, inspiration}\right)]\times 100$$

In addition, the abdominal adipose tissue area was quantified using single-slice abdominal CT scans obtained with the same CT scanner (Brilliance 64, Philips Healthcare). A 5-mm CT slice scan was acquired at the L4–L5 vertebral interspace, and VFA was measured by determining the mean values of all pixels within the range of − 190 to − 30 Hounsfield units. Scans were converted into a format compatible with commercial software (Extended Brilliance Workstation, Philips Healthcare).

### Other variables

Demographic characteristics, smoking and alcohol status, and medical conditions were obtained via questionnaires. Body mass index (BMI) was calculated as weight (kg) divided by height squared (m^2^). Fasting glucose, hemoglobin A1c, total cholesterol, triglyceride, high-density lipoprotein cholesterol, and high-sensitivity C-reactive protein levels were measured using standardized enzymatic methods in a commercial laboratory (Seoul Clinical Laboratories, Seoul, Korea). Low-density lipoprotein cholesterol levels were estimated using the Friedewald formula. Hypertension was defined as systolic blood pressure ≥ 140 mmHg, diastolic blood pressure ≥ 90 mmHg, or current use of antihypertensive medications. Type 2 diabetes was defined as fasting blood glucose level ≥ 126 mg/dL or use of insulin or oral hypoglycemic medications. Chronic obstructive pulmonary disease and asthma were defined as a history of medications and treatments for these conditions. Details for assessment of sleep-related parameters by questionnaires can be found in Supplementary information.

### Statistical analysis

The demographic characteristics of the study participants are presented as mean ± standard deviation and proportions, stratified according to severity of OSA (AHI < 5, 5 ≤ AHI < 15, 15 ≤ AHI) using the *χ*^2^ test or one-way analysis of variance, for categorical and continuous variables, respectively. The associations between the sleep/tracheal CT parameters and OSA severity were assessed using one-way analysis of covariance and post hoc Scheffe’s test. Multivariate linear regression analysis was performed to determine the effects of central airway collapse after adjustment for age, BMI, hypertension, type 2 diabetes, pack years of smoking, and inspiratory whole lung volume. All statistical analyses were performed using SAS software (version 9.4, SAS Institute).

## Results

### Descriptive analysis

Table 1 shows the demographic results according to OSA severity in men (*n* = 485, 53.8%) and women (*n* = 416, 46.2%). Of all participants, prevalence of mild OSA (5 ≤ AHI < 15) was similar in men and women (men, 33.2%; women, 31.0%), but men who had moderate-to-severe OSA (15 ≤ AHI) had it higher than women (men, 23.3%; women, 12.7%). In men, participants with moderate-to-severe OSA had higher BMI (26.10 ± 2.89 vs. 24.02 ± 2.46 kg/m^2^, *p* < 0.001) and visceral fat area (128.66 ± 48.50 vs. 98.99 ± 47.68 cm^2^, *p* < 0.001), and had higher prevalence of hypertension (58.4% vs. 46.0%, *p* = 0.03) and type 2 diabetes (48.7% vs. 28.4%, *p* < 0.001) than participants without OSA. However, age and lifestyles were not different among severity groups of OSA. Meanwhile, women with moderate-to-severe OSA were slightly older (64.53 ± 7.96 vs. 58.81 ± 6.03 years, *p* < 0.001), had higher BMI (26.52 ± 3.41 vs. 23.76 ± 2.76 kg/m^2^, *p* < 0.001) and visceral fat area (120.14 ± 47.00 vs. 77.45 ± 32.31 cm^2^, *p* < 0.001), and had higher prevalence of hypertension (66.0% vs. 31.2%, *p* < 0.001) and type 2 diabetes (50.9% vs. 20.5%, *p* < 0.001) than women without OSA. The distribution of the postmenopausal status was over 94% in both OSA and no OSA women.

**Table 1:** Comparison of clinical characteristics according to severity of obstructive sleep apnea in men and women (*N* = 901)

	Men (*N* = 485, 53.8%)										Women (*N* = 416, 46.2%)									
	No OSA (AHI < 5) (*N* = 211, 43.5%)			Mild OSA (5 ≤ AHI < 15) (*N* = 161, 33.2%)			Moderate-to-severe OSA (15 ≤ AHI) (*N* = 113, 23.3%)			*p* value	No OSA (AHI < 5) (*N* = 234, 56.3%)			Mild OSA (5 ≤ AHI < 15) (*N* = 129, 31.0%)			Moderate-to-severe OSA (15 ≤ AHI) (*N* = 53, 12.7%)			*p* value
Age (years)	59.51	±	6.96	60.16	±	6.35	60.89	±	7.70	0.23	58.81	±	6.03	61.12	±	7.60	64.53	±	7.96	< 0.001*^†‡^
Body mass index (kg/m^2^)	24.02	±	2.46	24.94	±	2.58	26.10	±	2.89	< 0.001^*†‡^	23.76	±	2.76	25.80	±	3.36	26.52	±	3.41	< 0.001*^†^
Waist circumference (cm)	84.69	±	7.43	87.70	±	7.39	90.32	±	7.20	< 0.001^*†‡^	78.48	±	7.34	84.31	±	8.43	87.53	±	8.26	< 0.001*^†‡^
Neck circumference (cm)	37.56	±	2.49	38.42	±	4.45	38.95	±	2.34	< 0.001^*†^	32.51	±	2.16	33.75	±	1.78	34.13	±	2.16	< 0.001*^†^
Visceral fat area (cm^2^)	98.99	±	47.68	111.56	±	52.59	128.66	±	48.50	< 0.001^*‡^	77.45	±	32.31	108.87	±	48.87	120.14	±	47.00	< 0.001*^†^
Fasting glucose (mg/dL)	98.65	±	21.34	102.31	±	23.36	107.85	±	27.47	0.004^†^	91.00	±	14.26	96.84	±	19.24	97.89	±	17.28	< 0.001*^†^
HbA1c (%)	5.75	±	0.69	5.92	±	1.04	6.16	±	0.92	< 0.001^†^	5.68	±	0.59	5.98	±	0.79	6.17	±	0.78	< 0.001*^†^
Total cholesterol (mg/dL)	190.10	±	35.28	183.86	±	35.37	182.18	±	39.07	0.11	203.31	±	34.72	196.33	±	37.07	185.09	±	38.56	0.003^†^
Triglyceride (mg/dL)	139.57	±	90.24	137.98	±	77.16	150.88	±	79.37	0.40	117.57	±	57.48	127.16	±	57.19	160.51	±	113.09	< 0.001^†‡^
HDL-cholesterol (mg/dL)	44.43	±	10.79	44.23	±	10.51	39.89	±	8.85	< 0.001^†‡^	51.00	±	12.75	48.56	±	11.83	44.30	±	11.62	0.001^†^
LDL-cholesterol (mg/dL)	118.39	±	31.28	111.91	±	30.94	112.65	±	35.58	0.11	128.79	±	32.40	122.34	±	32.68	109.22	±	33.09	< 0.001^†^
hsCRP (mg/dL)	1.41	±	2.42	1.13	±	1.41	1.68	±	3.58	0.19	1.21	±	2.27	1.42	±	1.97	1.02	±	0.87	0.44
Pack-years of smoking	18.97	±	18.45	18.09	±	17.58	20.24	±	22.41	0.66	0.14	±	1.63	0.38	±	3.26	0.11	±	0.82	0.59
Alcohol consumption (g/day)	18.75	±	30.85	16.74	±	24.71	13.84	±	28.83	0.37	1.34	±	4.44	0.80	±	3.73	1.94	±	6.49	0.31
Physical activity (MET/wk)	1003.36	±	1117.02	761.27	±	1038.27	1053.19	±	1279.63	0.06	749.07	±	841.51	695.85	±	798.55	566.04	±	610.18	0.32
Menopause, *n* (%)	-		-	-		-	-		-	-	221		(94.4)	123		(95.4)	52		(98.1)	0.53
Hypertension, *n* (%)	97		(46.0)	93		(57.8)	66		(58.4)	0.03^*†^	73		(31.2)	56		(43.4)	35		(66.0)	< 0.001^*†‡^
Type 2 diabetes, *n* (%)	60		(28.4)	59		(36.7)	55		(48.7)	0.001^†‡^	48		(20.5)	43		(33.3)	27		(50.9)	< 0.001^*†‡^
Asthma, *n* (%)	7		(3.3)	7		(4.4)	2		(1.8)	0.50	9		(3.9)	8		(6.2)	4		(7.6)	0.42
COPD, *n* (%)	79		(37.4)	64		(39.8)	28		(24.8)	0.03^†‡^	50		(21.4)	14		(10.9)	8		(15.1)	0.04^*^
Beck Depression Inventory (BDI)	5.27	±	5.27	5.99	±	6.23	5.39	±	5.40	0.45	7.80	±	6.44	7.79	±	6.23	7.51	±	6.30	0.95
Clinical depression (borderline to extreme, 17 ≤ BDI), *n* (%)	9		(4.3)	14		(8.7)	3		(2.7)	0.06	23		(10.0)	10		(7.9)	5		(9.4)	0.81
Chest CT parameters																				
Whole-lung volume (insp) (cc)	5709.47	±	854.00	5674.81	±	953.43	5652.01	±	868.46	0.85	4156.19	±	667.40	4063.64	±	691.79	3993.08	±	656.48	0.19
Whole-lung volume (exp) (cc)	2832.78	±	688.14	2698.19	±	560.99	2625.50	±	545.82	0.009^†^	2068.23	±	441.79	1961.32	±	451.74	1947.03	±	380.97	0.04^*^
Mean of tracheal length (mm)	117.40	±	11.33	117.03	±	11.62	119.00	±	11.40	0.34	106.54	±	11.10	107.18	±	11.89	105.65	±	10.00	0.69

Data are presented as *n* (%) or mean ± standard deviation, unless otherwise stated

*OSA*, obstructive sleep apnea; *HbA1c*, hemoglobin A1c; *HDL*, high-density lipoprotein; *LDL*, low-density lipoprotein; *hsCRP*, high-sensitivity C-reactive protein; *MET*, metabolic equivalent; *COPD*, chronic obstructive pulmonary disease

^*^p < 0.05, when comparing no OSA with mild OSA using the post hoc Scheffe test

^†^*p* < 0.05, when comparing no OSA with moderate-to-severe OSA using the post hoc Scheffe test

^‡^*p* < 0.05 when comparing mild OSA with moderate-to-severe OSA using the post hoc Scheffe test

In addition, both men and women with mild OSA or moderate-to-severe OSA showed lower expiratory whole-lung volume than participants with no OSA (men, *p* = 0.009; women, *p* = 0.04), but tracheal length did not differ among all participants with different OSA severities (Table 1).

### OSA severity and sleep parameters

Both men and women with moderate-to-severe OSA showed higher awakening index (7.47 ± 5.16 vs. 5.50 ± 2.73, *p* < 0.001 in men; 5.83 ± 5.23 vs. 4.16 ± 2.01, *p* < 0.001 in women) and lower oxygen saturation values than no OSA: the mean oxygen saturation (94.23 ± 1.43 vs. 95.63 ± 1.02, *p* < 0.001 in men; 94.30 ± 1.49 vs. 96.02 ± 1.11, *p* < 0.001 in women), the lowest oxygen saturation (79.71 ± 8.17 vs. 89.27 ± 6.55, *p* < 0.001 in men; 81.36 ± 4.64 vs. 89.67 ± 5.14, *p* < 0.001 in women), and percentage of oxygen saturation under 90% (5.40 ± 8.57 vs. 0.41 ± 1.27, *p* < 0.001 in men; 4.55 ± 8.37 vs. 0.13 ± 0.24, *p* < 0.001 in women). However, sleep quality, excessive daytime sleepiness, and insomnia severity according to OSA severity were not different in both men and women (Table 2).

**Table 2:** Comparison of sleep parameters according to severity of obstructive sleep apnea in men and women (*N* = 901)

	Men (*N* = 485, 53.8%)										Women (*N* = 416, 46.2%)									
	No OSA (AHI < 5) (*N* = 211, 43.5%)			Mild OSA (5 ≤ AHI < 15) (*N* = 161, 33.2%)			Moderate-to-severe OSA (15 ≤ AHI) (*N* = 113, 23.3%)			*p* value^§^	No OSA (AHI < 5) (*N* = 234, 56.3%)			Mild OSA (5 ≤ AHI < 15) (*N* = 129, 31.0%)			Moderate-to-severe OSA (15 ≤ AHI) (*N* = 53, 12.7%)			*p* value^§^
Total sleep time (min)	351.69	±	82.53	370.51	±	75.93	362.65	±	74.59	0.07	375.94	±	78.80	363.62	±	86.45	358.56	±	79.34	0.21
AHI (event/h of TST)	2.15	±	1.42	8.65	±	2.86	26.55	±	12.24	< 0.001*^†‡^	2.01	±	1.38	8.64	±	2.73	23.15	±	8.57	< 0.001*^†‡^
Supine AHI (event/h)	5.52	±	7.59	19.04	±	12.81	42.78	±	18.94	< 0.001*^†‡^	3.96	±	5.11	17.84	±	14.17	39.69	±	19.17	< 0.001*^†‡^
Non-supine AHI (event/h)	0.95	±	1.05	2.61	±	2.36	12.00	±	14.22	< 0.001^†‡^	0.64	±	0.86	2.94	±	3.58	10.21	±	10.14	< 0.001*^†‡^
Awakening index > 30 s (event/h)	5.50	±	2.73	6.10	±	3.28	7.47	±	5.16	< 0.001^†‡^	4.16	±	2.01	4.36	±	2.65	5.83	±	5.23	< 0.001^†‡^
Mean SaO2 (%)	95.63	±	1.02	95.18	±	1.13	94.23	±	1.43	< 0.001^†‡^	96.02	±	1.11	95.28	±	1.22	94.30	±	1.49	< 0.001^†‡^
Lowest SaO_2_ (%)	89.27	±	6.55	84.97	±	5.57	79.71	±	8.17	< 0.001*^†‡^	89.67	±	5.14	84.64	±	4.93	81.36	±	4.64	< 0.001*^†‡^
Oxygen Desaturation Index (event/h of TST)	1.90	±	1.35	7.54	±	2.89	24.30	±	11.86	< 0.001*^†‡^	1.87	±	1.26	7.96	±	2.75	21.95	±	8.45	< 0.001*^†‡^
SaO_2_ < 90% (min)	1.18	±	3.63	2.69	±	3.37	17.12	±	24.32	< 0.001^†‡^	0.48	±	0.80	3.27	±	6.59	16.61	±	31.03	< 0.001^†‡^
SaO_2_ < 90% (% TST)	0.41	±	1.27	0.75	±	0.96	5.40	±	8.57	< 0.001^†‡^	0.13	±	0.24	0.90	±	1.69	4.55	±	8.37	< 0.001^†‡^
PSQI global score	3.33	±	2.67	3.49	±	2.30	3.37	±	2.61	0.82	4.35	±	2.91	4.46	±	3.20	4.57	±	3.11	0.87
Excessive daytime sleepiness (ESS ≥ 12)	5		(2.4)	5		(3.1)	3		(2.8)	0.92	9		(3.9)	6		(4.7)	4		(7.6)	0.52
Insomnia severity index (ISI)	6.38	±	5.11	6.61	±	5.00	6.58	±	5.13	0.91	7.16	±	4.88	7.43	±	4.99	8.29	±	6.61	0.38
Subthreshold insomnia (8 ≤ ISI < 15), *n* (%)	60		(31.4)	52		(35.4)	36		(34.0)	0.84	77		(34.8)	43		(35.8)	17		(35.4)	0.28
Clinical insomnia (moderate to severe) (15 ≤ ISI), *n* (%)	13		(6.8)	7		(4.8)	8		(7.6)		19		(8.6)	11		(9.2)	9		(18.8)	

Data are presented as *n* (%) or mean ± standard deviation, unless otherwise stated

*OSA*, obstructive sleep apnea; *AHI*, apnea–hypopnea index; *SaO*_*2*_, oxygen saturation; *TST*, total sleep time; *ESS*, Epworth Sleepiness Scale

^*^p < 0.05, when comparing no OSA with mild OSA using the post hoc Scheffe test

^†^*p* < 0.05, when comparing no OSA with moderate-to-severe OSA using the post hoc Scheffe test

^‡^*p* < 0.05 when comparing mild OSA with moderate-to-severe OSA using the post hoc Scheffe test

### OSA severity and tracheal CT parameters

A comparison of tracheal CT parameters during inspiration and expiration in men and women with different OSA severities is shown in Table 3. After adjusting for age, BMI, hypertension, type 2 diabetes, pack-year of smoking, and inspiratory whole-lung volume, the percentage of expiratory lumen area reduction was increased in women with moderate-to-severe OSA compared with those without OSA (21.89 ± 12.28 vs. 17.74 ± 8.43, *p* = 0.02), but men were not statistically significant different according to OSA severity. In women, the percentage of expiratory lumen diameter and lumen perimeter reductions were also larger in the moderate-to-severe OSA group than in the no OSA group (both *p* values = 0.02), but not men.

**Table 3:** Associations of tracheal computed tomography parameters and OSA severity

	Men (*N *= 485, 53.8%)				Women (*N *= 416, 46.2%)			
	No OSA (AHI < 5) (*N *= 211, 43.5%)	Mild OSA (5 ≤ AHI < 15) (*N *= 161, 33.2%)	Moderate-to-severe OSA (15 AHI) (*N *= 113, 23.3%)	*p* value^§^	No OSA (AHI < 5 (*N*=234, 56.3%)	Mild OSA (5 ≤ AHI < 15) (*N *= 129, 31.0%)	Moderate-to-severe OSA (15 ≤ AHI) (*N *= 53, 12.7%)	*p* value^§^
Inspiration, mean ± SD								
Mean of lumen area (mm^2^)	282.69 ± 43.64	282.58 ± 42.83	277.54 ± 43.28	0.87	188.52 ± 31.85	187.72 ± 29.32	186.36 ± 30.62	0.91
Mean of lumen diameter (mm)	18.84 ± 1.46	18.84 ± 1.43	18.65 ± 1.46	0.84	15.38 ± 1.31	15.35 ± 1.21	15.29 ± 1.26	0.89
Mean of lumen perimeter (mm)	59.43 ± 4.58	59.42 ± 4.49	58.88 ± 4.57	0.86	48.50 ± 4.12	48.42 ± 3.76	48.23 ± 3.96	0.87
Mean of wall area (mm^2^)	177.83 ± 16.67	178.04 ± 16.03	177.54 ± 17.22	0.95	142.02 ± 13.78	141.59 ± 12.44	142.34 ± 13.29	0.86
Mean of wall thickness (mm)	2.58 ± 0.11	2.59 ± 0.11	2.59 ± 0.13	0.98	2.49 ± 0.10	2.49 ± 0.10	2.50 ± 0.11	0.64
Expiration, mean ± SD								
Mean of lumen area (mm^2^)	232.98 ± 36.50	232.66 ± 33.86	222.86 ± 33.26	0.16	153.82 ± 22.95	148.31 ± 25.30	144.68 ± 29.12	0.35
Mean of lumen diameter (mm)	17.11 ± 1.36	17.09 ± 1.25	16.72 ± 1.26	0.16	13.91 ± 1.04	13.63 ± 1.18	13.44 ± 1.39	0.25
Mean of lumen perimeter (mm)	53.94 ± 4.23	53.93 ± 3.92	52.77 ± 3.94	0.16	43.84 ± 3.27	43.01 ± 3.69	42.43 ± 4.31	0.29
Mean of wall area (mm^2^)	167.79 ± 14.83	167.47 ± 16.06	164.62 ± 14.70	0.16	130.34 ± 10.49	130.17 ± 12.44	127.55 ± 14.20	0.38
Mean of wall thickness (mm)	2.64 ± 0.11	2.65 ± 0.13	2.64 ± 0.12	0.59	2.50 ± 0.10	2.52 ± 0.11	2.51 ± 0.14	0.22
Percentage of expiratory reduction in tracheal CT parameters (Expiration/Inspiration) (%), mean ± SD								
Mean of lumen area (mm^2^)	17.14 ± 8.89	17.20 ± 8.13	19.14 ± 8.74	0.18	17.74 ± 8.43	20.65 ± 8.89	21.89 ± 12.28	0.02*†
Mean of lumen diameter (mm)	9.12 ± 5.07	9.16 ± 4.63	10.22 ± 4.99	0.20	9.42 ± 4.68	11.07 ± 5.34	11.99 ± 7.35	0.02*†
Mean of lumen perimeter (mm)	9.11 ± 4.99	9.12 ± 4.54	10.22 ± 4.99	0.18	9.42 ± 4.67	11.08 ± 5.22	11.90 ± 7.16	0.02*†
Mean of wall area (mm^2^)	5.90 ± 5.49	6.62 ± 5.67	7.55 ± 6.58	0.15	8.57 ± 6.02	8.56 ± 6.60	10.27 ± 7.81	0.33
Mean of wall thickness (mm)	0.48 ± 1.29	0.87 ± 1.85	1.42 ± 1.89	0.01†	1.44 ± 1.85	1.40 ± 2.45	1.80 ± 2.34	0.55

*OSA* obstructive sleep apnoea, *AHI* apnoea-hypopnea index, *SD* standard deviation, *insp* inspiration, *exp* expiration

^*^p < 0.05 when comparing no OSA with mild OSA using the post hoc Scheffe test

^†^*p* < 0.05 when comparing no OSA with moderate-to-severe OSA using the post hoc Scheffe test

^‡^*p* < 0.05 when comparing mild OSA with moderate-to-severe OSA using the post hoc Scheffe test

^§^*p* values for one-way analysis of covariance including age, body mass index, hypertension, type 2 diabetes, pack-year of smoking, and inspiratory whole lung volume

In women, more severe AHI was associated with a larger percentage of expiratory tracheal collapse through all of lumen area, diameter, and perimeter reductions after adjusting covariates (all *p* values < 0.01) (Table 4). In multivariate analysis, mild OSA and moderate-to-severe OSA were associated with the percentage of expiratory lumen area reduction after adjusting for age, sex, BMI, hypertension, type 2 diabetes, alcohol consumption, pack-years of smoking, and inspiratory whole-lung volume (mild OSA: *β* = 2.45%, standard error (SE) = 1.05, *p* = 0.02; moderate-to-severe OSA: *β* = 3.30%, SE = 1.51, *p* = 0.03). In addition, the percentages of expiratory lumen diameter and lumen perimeter reductions also showed a positive correlation with both mild OSA (*β* = 1.38%, SE = 0.61, *p* = 0.02; and *β* = 1.38%, SE = 0.60, *p* = 0.02, respectively) and moderate-to-severe OSA (*β* = 2.05%, SE = 0.87, *p* = 0.02; and *β* = 1.97%, SE = 0.86, *p* = 0.02, respectively), whereas men had no differences all of percentage of expiratory reduction in tracheal CT parameters except for percentage of expiratory wall thickness reduction (moderate-to-severe OSA: *β* = 0.72%, SE = 0.24, *p* = 0.003).

**Table 4:** Multivariate linear regression analysis of the relationship between OSA severity and percentage of expiratory reduction in tracheal computed tomography parameters by sex

Models	Relationship to AHI		Comparison of OSA group					
	AHI		No OSA (AHI < 5)	Mild OSA (5 ≤ AHI < 15)		Moderate-to-severe OSA (15 ≤ AHI)		*P*_trend_*
	*β* (SE)	*p* value*		*β* (SE)	*p* value*	*β* (SE)	*p* value*	
Men (*N *= 485, 53.8%)								
Percentage of expiratory tracheal collapse (Exp/insp) (lumen area reduction)	0.055 (0.036)	0.13	Ref.	–0.24 (0.90)	0.79	1.65 (1.05)	0.12	0.17
Percentage of expiratory lumen diameter reduction (Exp/insp)	0.031 (0.021)	0.13	Ref.	–0.13 (0.51)	0.81	0.91 (0.60)	0.13	0.19
Percentage of expiratory lumen perimeter reduction (Exp/insp)	0.031 (0.021)	0.13	Ref.	–0.15 (0.51)	0.77	0.92 (0.59)	0.12	0.18
Percentage of expiratory wall area reduction (Exp/insp)	0.057 (0.025)	0.02	Ref.	0.56 (0.62)	0.36	1.40 (0.72)	0.05	0.05
Percentage of expiratory wall thickness reduction (Exp/insp)	0.019 (0.008)	0.02	Ref.	0.31 (0.20)	0.11	0.72 (0.24)	0.003	0.003
Women (*N *= 416, 46.2%)								
Percentage of expiratory tracheal collapse (Exp/insp) (lumen area reduction)	0.101 (0.064)	0.12	Ref.	2.45 (1.05)	0.02	3.30 (1.51)	0.03	0.008
Percentage of expiratory lumen diameter reduction (Exp/insp)	0.064 (0.037)	0.09	Ref.	1.38 (0.61)	0.02	2.05 (0.87)	0.02	0.006
Percentage of expiratory lumen perimeter reduction (Exp/insp)	0.060 (0.037)	0.11	Ref.	1.38 (0.60)	0.02	1.97 (0.86)	0.02	0.007
Percentage of expiratory wall area reduction (Exp/insp)	0.021 (0.046)	0.64	Ref.	–0.42 (0.75)	0.58	1.18 (1.07)	0.27	0.50
Percentage of expiratory wall thickness reduction (Exp/insp)	–0.005 (0.016)	0.74	Ref.	–0.19 (0.27)	0.48	0.21 (0.37)	0.58	0.86

*OSA* obstructive sleep apnoea, *AHI* apnoea-hypopnea index, *SE* standard error, *exp* expiration, *insp* inspiration, *Ref* reference

*Adjusted for age, body mass index, hypertension, type 2 diabetes, pack-year of smoking, and inspiratory whole lung volume

### Joint effects of OSA severity and sex on tracheal CT parameters

**Fig. 3:**
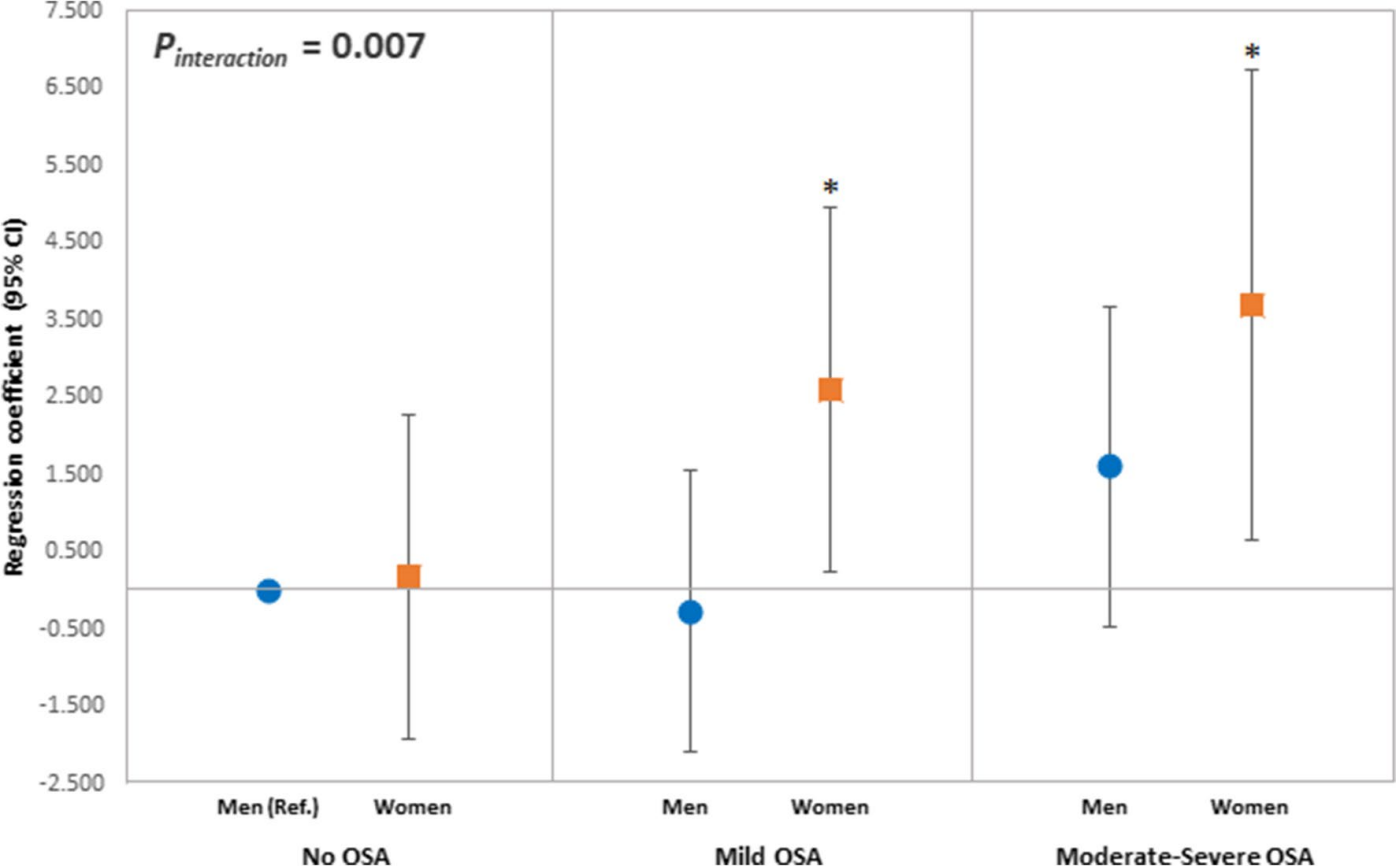
Association between OSA severity and percentage of tracheal collapse according by sex. All *p* values were adjusted for age, body mass index, hypertension, type 2 diabetes, pack-years of smoking, and inspiratory whole lung volume. Error bars represent 95% CI. *p < 0.05. OSA, obstructive sleep apnea; Ref., reference; CI, confidence interval

As shown in Fig. 3, women with both mild OSA and moderate-to-severe OSA had higher expiratory tracheal collapse (mild OSA, *β* = 2.61% [95% CI: 0.24, 4.97], *p* = 0.03; moderate-to-severe OSA, *β* = 3.68% [95% CI: 0.64, 6.72], *p* = 0.02) than men without OSA. Meanwhile, OSA severity in men was not significantly different compared to no OSA in men. In addition, a moderate effect of sex was observed (*p*_interaction_ = 0.007).

## Discussion

The principal finding in a present study was that differences according to sex in degree of the expiratory central airway collapse on severity of OSA, especially moderate-to-severe OSA. We found that in moderate-to-severe OSA, women had greater collapsibility of expiratory lumen structures including area, diameter, and perimeter than men after accounting for age, BMI, hypertension, diabetes, smoking burden, and lung volume, whereas men with moderate-to-severe OSA had greater reduction of expiratory wall thickness compared with equivalent severity of OSA in women.

Recent advances in quantitative imaging techniques have shown meaningful differences in both upper airway soft tissue and craniofacial bony structures in patients with OSA. Studies involving nasal pharyngoscopy, CT, or magnetic resonance imaging have revealed that closure usually occurs in one or multiple sites within the pharyngeal region in most individuals with OSA, and this region is also smaller in patients of OSA than in controls even during wakefulness [28, 29]. Specific skeletal conditions coexist with particular soft tissue features in OSA patients. Bacon et al reported that OSA patients have smaller sizes of craniofacial and pharyngeal bony structures including a short mandibular length, posterior facial compression, inferiorly positioned hyoid bone, and a retroposed maxilla and mandible, all of which compromise the pharyngeal space [30, 31]. Therefore, treatments with continuous positive airway pressure, mandibular advancement, and weight loss have all been shown to increase the lateral pharyngeal dimensions [28, 32].

One potential explanation for gender dissimilarities in degree of ECAC on OSA severity is the anatomical difference of the upper airway between genders. The thinner airway walls in women are compatible with findings that women appear to have more acclimatized airways with greater collapsibility than men [33]. In addition, the lumen size of the airway, with direct impacts for higher airway resistance, was lower in women compared with men even with adjustment for the same covariates. Prior CT studies also have reported smaller luminal diameter in the central airways in women [34, 35]. In our data, we also have shown that women have smaller airway lumen structures including area, diameter, and perimeter than men after accounting for age, BMI, hypertension, diabetes, pack-year of smoking, and inspiratory whole-lung volume (online supplemental Table E1). The trachea expands during inhalation and narrows during exhalation of breath because of the pressure gradient across the airway wall. The lumen diameter of the airway is affected by the transmural airway pressure gradient, and the differences between the pressures inside the lumen of the airway and the pressure surrounding the airway wall [19]. According to the collapsible tube model, an increase in *P*_tissue_ decreases the cross-sectional luminal area of the tube. In addition, the pharyngeal airway shares space with the soft tissues within the maxillomandibular bony structure. The balance between the size of the bony structure and the amount of soft tissue may affect the airway space and *P*_tissue_ [36]. Bhatt and colleagues suggest that the lower space conferred by smaller airways may influence women to develop airflow limitation primarily. That is, the smaller airways in women can result in greater airway resistance and more turbulent airflow, and thus place a greater ventilatory restriction. These inherent features of airway in women can be associated with having more vulnerability of dyspnea and lower functional capacity in women than in men [31].

Furthermore, differences in fat and/or muscle distribution and tones between men and women may partly explain gender differences of luminal structures because *P*_tissue_ can be increased by anatomical unbalance of the upper airway due to the deposition of fat surrounding the airway within the maxillomandibular bony structure [17]. Specifically, menopause in women changes body fat and pharyngeal dilator muscle activity, and loss of female sex hormones has been proposed as potential contributing factors because female sex hormones are also known as having a protective effect on upper airway patency [9]. During menopausal transition, increased collapsibility of the soft tissue and arousals predispose to respiratory imbalance and exacerbate upper airway obstruction. Prevalence of OSA in women increases twofold after menopause independently of age and BMI [37, 38]. An enlargement in the amount of soft tissue within the bony structure or a contraction in the size of the bony structure would limit the space available for the airway and result in narrowing of the airway and an increase in *P*_tissue_ [19]. In addition, when the activity of muscles expanding the upper airway decreases, the pharynx is intrinsically collapsible during sleep [39].

The strengths of this study were the inclusion of participants from the general population, and the use of OSA severity objectively measured using PSG and airway parameters noninvasively measured using tomographic imaging. However, some limitations need to be considered when interpreting our results. Some participants have missing tracheal measurement data. Fortunately, there were no differences in OSA severity, age, lifestyle, cardiovascular conditions, hypertension, and diabetes between the included and excluded participants (online supplemental Table E2). Additionally, we used a cross-sectional study design. Thus, further studies with a long-term follow-up are needed to determine the causal relationship between airway collapse and OSA.

In conclusion, there are differences in tracheal dynamic collapse as quantified at CT on OSA severity between men and women. The expiratory dynamic tracheal collapsibility is independently associated with moderate-to-severe OSA in women than in men after accounting for covariates. Differences of pharyngeal structures and inherent features of airways by genders may affect the dissimilarities in vulnerability to sleep apnea between men and women. Our findings might contribute to understanding disease progression and to looking for treatment options to provide best outcomes depending on men and women with OSA.

### Supplementary Information

Below is the link to the electronic supplementary material.

Supplementary file1 (PDF 650 KB)

## Abbreviations

AHI: Apnea–hypopnea index
BDI: Beck Depression Inventory
BMI: Body mass index
CT: Computed tomography
ECAC: Expiratory central airway collapse
ISI: Insomnia Severity Index
OSA: Obstructive sleep apnea OSA
PSG: Polysomnography
PSQI: Pittsburgh Sleep Quality Index
VFA: Visceral fat area

## Declarations

### Guarantor

The scientific guarantor of this publication is Dr. Chol Shin.

### Statistics and biometry

Dr. Robert D. Abbott kindly provided statistical advice for this manuscript. In addition, one of the authors has significant statistical expertise (Dr. Siddiquee).

### Study subjects or cohorts overlap

We have previously reported using participants of the Korean Genome and Epidemiology Study [1]. This prior study dealt with the association between OSA and the presence of subclinical systemic atherosclerosis including tomographic quantification of the calcium burden.

1. Kim S, Lee KY, Kim NH, et al Relationship of obstructive sleep apnoea severity and subclinical systemic atherosclerosis. Eur Respir J. 2020;55(2):1900959.

### Methodology

• prospective

• cross-sectional study

• performed at one institution
