# Supplementary material for: Gender differences in association between expiratory dynamic airway collapse and severity of obstructive sleep apnea
Source: Eur Radiol. 2023 Nov 14;34(6):3730–41. doi: 10.1007/s00330-023-10322-x (PMC11166772; doi:10.1007/s00330-023-10322-x)
Supplement: Supplementary file 1 — Supplementary file1 (PDF 650 KB) [file 330_2023_10322_MOESM1_ESM.pdf]

# Supplemental Material

**Title:** Gender differences in association between expiratory dynamic airway collapse and severity of obstructive sleep apnea

**Authors:** Soriul Kim[1,2], Ki Yeol Lee[3,4*], Ali Tanweer Siddiquee[1], Hyeon-Jin Kim[5], Hye Ryeong Nam[1], Chang Seop Ko[3], Nan Hee Kim[6], and Chol Shin[1,7,8*]

## Supplemental Materials and Methods

### *Study design and population*

All study participants were part of the 2001-2002 Korean Genome and Epidemiology Study, which is an ongoing prospective investigation. Detailed information on participant recruitment is available elsewhere [1-3]. Briefly, a total of 5012 participants from Ansan, South Korea, were examined at baseline between 2001 and 2002. The cohort participants had a questionnaire-based interview and health examination, and biospecimens were collected by health professionals. Blood samples were collected after a fasting period of at least 8h. The questionnaire included in demographic characteristics, medical history, lifestyle, and sleep-related parameters. The health examination comprised anthropometric and clinical evaluations, including chest CT. Follow-up examinations were performed biennially during scheduled site visits.

### *Polysomnography*

An oxygen desaturation event was detected when oxygen saturation dropped by at least 4%. All saturation values <50% were excluded as artifact values and were not considered as part of the desaturation event. An apnea event was detected if both of the following criteria were met: (i) a drop in the peak signal excursion by ≥90% of the pre-event baseline (reference amplitude) was observed and (ii) the duration of the ≥90% drop in the sensor signal was ≥10 s. In addition, a hypopnea event was detected if all of the following criteria were met: (i) the peak signal excursion dropped by ≥30% of the reference amplitude; (ii) the duration of the ≥30% drop in signal excursion was ≥10 s; and (iii) a ≥3% oxygen desaturation from the reference amplitude occurred or the event was associated with an

arousal. The reference amplitude was calculated as the mean value of the peak amplitudes in the period of 100 s preceding the event.

***Assessment for sleep-related parameters by questionnaires***

Insomnia severity was measured using the Insomnia Severity Index (ISI). The ISI is a seven-item self-administered questionnaire assessing the nature, severity and impact of insomnia in adults over the past 2 weeks. A 5-point Likert scale on a 0-4 point is used to rate each item, and the total score ranges from 0 to 28. Severity levels is categorized as no clinically significant insomnia (0 to 7 points), subthreshold insomnia (8 to 14 points), and moderate to severe insomnia (15 points or more) [4]. In addition, we measured depressive symptoms and daytime sleepiness. Depressive symptoms were measured using the Beck Depression Inventory (BDI). The BDI was developed to assess the type and degree of depression, based on symptoms of depression. The questionnaire contains 21 questions, and each item consists of four statements describing increasing intensities of symptoms of depression (total score ranges: 0-63); higher scores reflect more severe of depressive symptoms [5]. Daytime sleepiness was measured using the Epworth Sleepiness Scale, a validated eight-item questionnaire that assessed excessive daytime sleepiness. A sum score is calculated (range: 0-24), with lower scores indicating less sleepiness [6]. Sleep quality was assessed via the Pittsburgh Sleep Quality Index (PSQI), which is a widely used and well-validated 19-item questionnaire that measures sleep quality in adults [7]. It provides a global PSQI score (rages: 0-21), which consists of seven sub scores, and higher PSQI global score indicates poorer sleep.

**References**

1. Kim S, Lee KY, Kim NH, *et al.* Relationship of obstructive sleep apnoea severity and subclinical systemic atherosclerosis. *Eur Respir J.* 2020;55(2):1900959.

2. Shin C, Kim JY, Kim JY, *et al.* Association of habitual snoring with glucose and insulin metabolism in nonobese Korean adult men. *Am J Respir Crit Care Med.* 2005; 171: 287–291.

3. Baik I, Kim J, Abbott RD, *et al.* Association of snoring with chronic bronchitis. *Arch Intern Med.* 2008;168:167–173.

4. Bastien CH, Vallières A, Morin CM. Validation of the Insomnia Severity Index as an outcome measure for insomnia research. Sleep Med. 2001;2(4):297-307.

5. Suh S, Yang HC, Fairholme CP, et al., Who is at risk for having persistent insomnia symptoms? A longitudinal study in the general population in Korea. Sleep Med. 2014;15(2):180-6.

6. Kim H, Thomas RJ, Yun CH, et al., Association of Mild Obstructive Sleep Apnea With Cognitive Performance, Excessive Daytime Sleepiness, and Quality of Life in the General Population: The Korean Genome and Epidemiology Study (KoGES). Sleep. 2017;40(5).

7. Buysse DJ, Reynolds CF 3rd, Monk TH, Berman SR, Kupfer DJ. The Pittsburgh Sleep Quality Index: a new instrument for psychiatric practice and research. Psychiatry Res. 1989;28(2):193-213.

**Figure E1.** Methods for analysis of trachea. (A) Cross-section images were acquired from 10 locations. (B) Method for 120 profiles from each sample to measure the wall thickness of the tracheal wall. (C) Method of measuring the diameter of the airway.

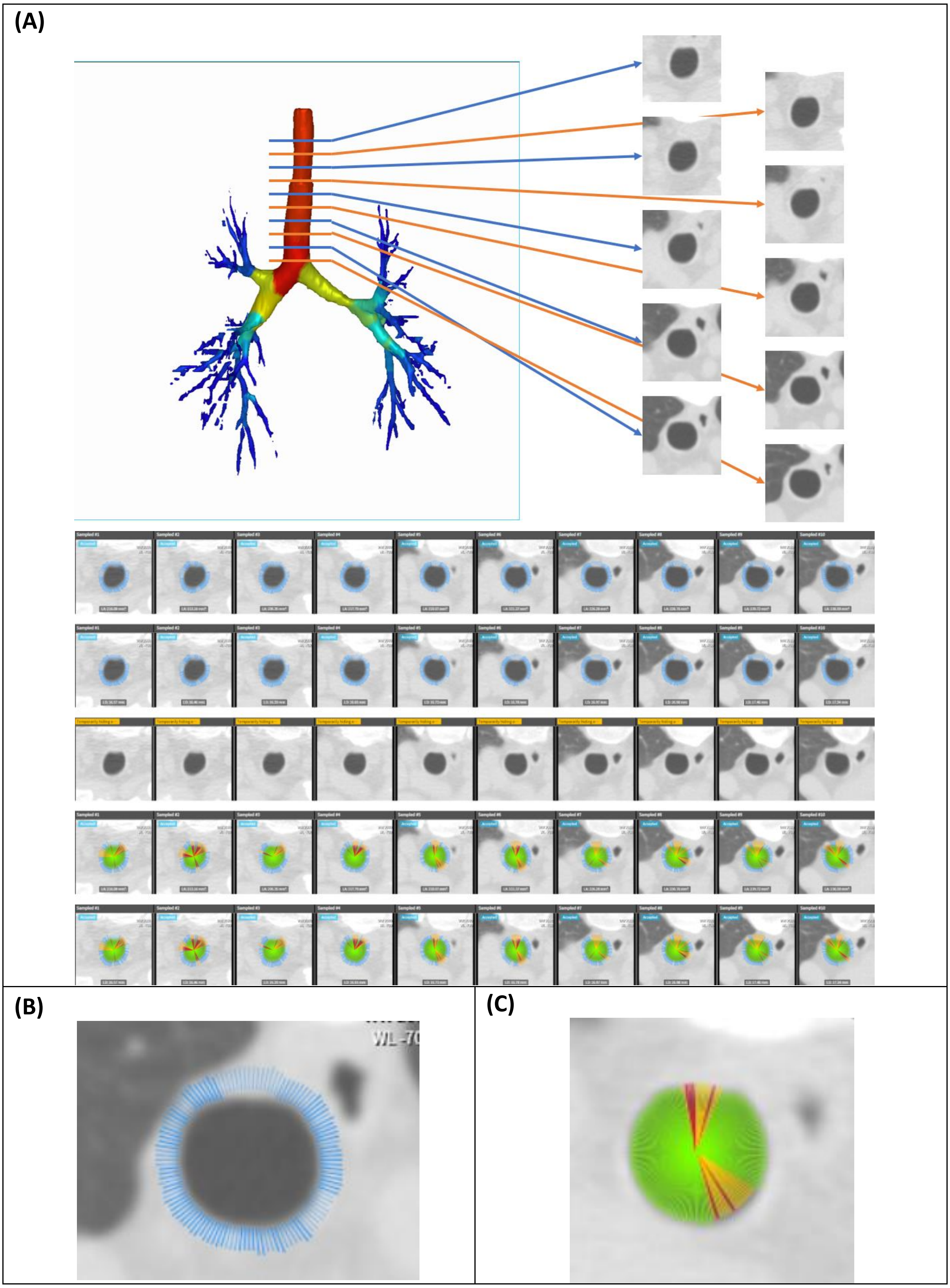

**Table E1. Comparison of tracheal computed tomography parameters between men and women**

| Variables | Men (n=485, 53.8%) | | | Women (n=416, 46.2%) | | | p-value* |
|---|---|---|---|---|---|---|---|
| Mean of tracheal length (mm) | 117.65 | ± | 11.45 | 106.63 | ± | 11.20 | <0.001 |
| Inspiration, mean±SD | | | | | | | |
| Mean of lumen area (mm²) | 281.45 | ± | 43.25 | 188.00 | ± | 30.87 | <0.001 |
| Mean of lumen diameter (mm) | 18.80 | ± | 1.45 | 15.36 | ± | 1.27 | <0.001 |
| Mean of lumen perimeter (mm) | 59.30 | ± | 4.55 | 48.44 | ± | 3.98 | <0.001 |
| Mean of wall area (mm²) | 177.83 | ± | 16.56 | 141.92 | ± | 13.28 | <0.001 |
| Mean of wall thickness (mm) | 2.59 | ± | 0.12 | 2.49 | ± | 0.10 | <0.001 |
| Expiration, mean±SD | | | | | | | |
| Mean of lumen area (mm²) | 230.52 | ± | 35.08 | 150.95 | ± | 24.72 | <0.001 |
| Mean of lumen diameter (mm) | 17.01 | ± | 1.31 | 13.76 | ± | 1.15 | <0.001 |
| Mean of lumen perimeter (mm) | 53.67 | ± | 4.09 | 43.41 | ± | 3.58 | <0.001 |
| Mean of wall area (mm²) | 166.94 | ± | 15.24 | 129.93 | ± | 11.65 | <0.001 |
| Mean of wall thickness (mm) | 2.64 | ± | 0.12 | 2.51 | ± | 0.11 | <0.001 |
| Percentage of expiratory reduction in tracheal CT parameters (Expiration/Inspiration) (%), mean±SD | | | | | | | |
| Mean of lumen area | 17.63 | ± | 8.64 | 19.17 | ± | 9.27 | 0.20 |
| Mean of lumen diameter | 9.39 | ± | 4.92 | 10.26 | ± | 5.37 | 0.23 |
| Mean of lumen perimeter | 9.37 | ± | 4.86 | 10.25 | ± | 5.29 | 0.21 |
| Mean of wall area | 6.52 | ± | 5.84 | 8.79 | ± | 6.47 | <0.001 |
| Mean of wall thickness | 0.81 | ± | 1.66 | 1.47 | ± | 2.10 | <0.001 |

*P-values for one-way analysis of covariance including age, body mass index, hypertension, type 2 diabetes, pack-years of smoking, and inspiratory whole lung volume.

**Table E2. Comparison of clinical characteristics between included and excluded participants**

| Variables | Included participants (N=901) | | | Excluded participants who underwent CT examination but with missing tracheal measurement data (N=1477) | | | p-value |
|---|---|---|---|---|---|---|---|
| Age (years) | 60.1 | ± | 7.0 | 60.7 | ± | 7.0 | 0.06 |
| Men, n (%) | 485 | | (53.8) | 702 | | (47.5) | 0.003 |
| Body mass index (kg/m$^2$) | 24.8 | ± | 3.0 | 24.5 | ± | 2.9 | 0.04 |
| Fasting glucose (mg/dL) | 98.2 | ± | 21.2 | 97.9 | ± | 21.9 | 0.74 |
| HbA1c (%) | 5.9 | ± | 0.8 | 5.8 | ± | 0.9 | 0.29 |
| Total cholesterol (mg/dL) | 192.0 | ± | 36.9 | 192.6 | ± | 36.8 | 0.69 |
| Triglyceride (mg/dL) | 134.4 | ± | 77.2 | 137.9 | ± | 90.5 | 0.33 |
| HDL-cholesterol (mg/dL) | 46.1 | ± | 11.8 | 46.7 | ± | 11.6 | 0.23 |
| LDL-cholesterol (mg/dL) | 119.3 | ± | 33.0 | 119.5 | ± | 32.7 | 0.91 |
| hsCRP (mg/dL) | 1.3 | ± | 2.3 | 1.4 | ± | 3.2 | 0.54 |
| Pack-years of smoking | 10.3 | ± | 16.9 | 9.6 | ± | 17.1 | 0.32 |
| Alcohol consumption (g/day) | 9.5 | ± | 22.4 | 7.6 | ± | 17.8 | 0.04 |
| Physical activity (MET/wk) | 830.6 | ± | 1002.3 | 823.7 | ± | 1002.3 | 0.87 |
| Hypertension, n (%) | 420 | | (46.6) | 692 | | (46.9) | 0.91 |
| Type 2 Diabetes, n (%) | 292 | | (32.4) | 482 | | (32.6) | 0.91 |
| Asthma, n (%) | 37 | | (4.1) | 43 | | (2.9) | 0.12 |
| COPD, n (%) | 243 | | (27.0) | 335 | | (22.7) | 0.02 |
| **Polysomnographic recordings** | | | | | | | |
| AHI (event/h of TST) | 8.5 | ± | 10.0 | 8.5 | ± | 10.3 | 0.98 |
| OSA, n (%) | | | | | | | 0.74 |
| No OSA | 445 | | (49.4) | 737 | | (49.9) | |
| Mild OSA | 290 | | (32.2) | 486 | | (32.9) | |
| Moderate-to-severe OSA | 166 | | (18.4) | 254 | | (17.2) | |

Data are presented as n (%) or mean ± standard deviation, unless otherwise stated.

CT, computed tomography; OSA, obstructive sleep apnea; AHI, apnoea-hypopnea index; $SaO_2$, oxygen saturation; HbA1c, hemoglobin A1c; HDL, high-density lipoprotein; LDL, low-density lipoprotein; hsCRP, high-sensitivity C-reactive protein; MET, metabolic equivalent; COPD, chronic obstructive pulmonary disease; TST, total sleep time.

**Table E3. Multivariate linear regression analysis of the relationship between OSA severity and percentage of expiratory reduction in tracheal CT parameters**

| Models | Relationship to AHI | | Comparison of OSA group | | | | | | | | |
|---|---|---|---|---|---|---|---|---|---|---|
| | AHI | | No OSA (AHI<5) | Mild OSA (5≤AHI<15) | | | Moderate-to-severe OSA (15≤AHI) | | | $P_{trend}$ |
| | β (SE) | P-value | | β | (SE) | P-value | β | (SE) | P-value | |
| **Percentage of expiratory tracheal collapse (Exp/insp) (lumen area reduction)** | | | | | | | | | | |
| Model 1 | 0.093 (0.030) | 0.002 | Ref. | 1.48 | (0.67) | 0.03 | 3.04 | (0.83) | <0.001 | <0.001 |
| Model 2 | 0.059 (0.032) | 0.06 | Ref. | 1.01 | (0.69) | 0.15 | 2.25 | (0.87) | 0.009 | 0.008 |
| Model 3 | 0.068 (0.032) | 0.03 | Ref. | 0.99 | (0.69) | 0.15 | 2.38 | (0.87) | 0.006 | 0.006 |
| **Percentage of expiratory lumen diameter reduction (Exp/insp)** | | | | | | | | | | |
| Model 1 | 0.055 (0.017) | 0.002 | Ref. | 0.84 | (0.39) | 0.03 | 1.77 | (0.48) | <0.001 | <0.001 |
| Model 2 | 0.036 (0.018) | 0.05 | Ref. | 0.58 | (0.40) | 0.15 | 1.33 | (0.50) | 0.007 | 0.007 |
| Model 3 | 0.040 (0.018) | 0.03 | Ref. | 0.56 | (0.39) | 0.15 | 1.40 | (0.50) | 0.005 | 0.005 |
| **Percentage of expiratory lumen perimeter reduction (Exp/insp)** | | | | | | | | | | |
| Model 1 | 0.053 (0.017) | 0.002 | Ref. | 0.82 | (0.38) | 0.03 | 1.74 | (0.47) | <0.001 | <0.001 |
| Model 2 | 0.034 (0.018) | 0.06 | Ref. | 0.56 | (0.39) | 0.16 | 1.30 | (0.49) | 0.008 | 0.008 |
| Model 3 | 0.039 (0.018) | 0.03 | Ref. | 0.55 | (0.39) | 0.16 | 1.37 | (0.49) | 0.005 | 0.005 |

OSA, obstructive sleep apnoea; AHI, apnoea-hypopnea index; SE, standard error; exp, expiration; insp, inspiration; Ref. reference.
Model 1: adjusted for age and sex. Model 2: adjusted for age, sex, and body mass index. Model 3: adjusted for age, sex, body mass index, hypertension, type 2 diabetes, pack-years of smoking, and inspiratory whole lung volume.
